# The complete chloroplast genome of the highly poisonous plant *Cerbera manghas* L. (Apocynaceae)

**DOI:** 10.1080/23802359.2020.1794994

**Published:** 2020-07-30

**Authors:** Miao Liao, Xue-Fen Wei, Hai-Ping Ding, Guang-Da Tang

**Affiliations:** a College of Forestry and Landscape Architecture, South China Agricultural University, Guangzhou, China; b College of Life Sciences, Shandong Agricultural University, Taian, China; c Henry Fok College of Biology and Agriculture, Shaoguan University, Shaoguan, China

**Keywords:** *Cerbera manghas*, plastome genome, Apocynaceae, Plumerieae

## Abstract

The complete chloroplast genome of *Cerber amanghas* L., a species of the tribe Plumerieae of the family Apocynaceae, is determined for the first time here. The chloroplast genome is 154,428 bp long, containing a large single-copy region (LSC) of 85,138 bp and a small single-copy region (SSC) of 17,390 bp, which are separated by a pair of 25,950 bp long inverted repeat regions (IRs). It encodes a total of 115 genes, including 81 unique protein-coding genes, 30 unique tRNA genes, and 4 unique rRNA genes.Phylogenetic analysis revealed that *C.manghas* is a member of the paraphyletic tribe Plumerieae of Apocynaceae and is closely related to *Thevetia peruviana.*

*Cerbera* belongs to the tribe of Plumerieae (Apocynaceae), small trees, sometimes shrubs, distributed in Madagascar and the Seychelles and from Japan (Ryukus) to Australia (Queensland) and Pitcairn (Endress et al. 2018). *Cerbera manghas*, the type species of the genus, grows by the sea, often is used as a landscape tree species inland (Agendae Academiae Sinicae Edita 1977). In addition, it has been used as the traditional medicine in some regions (Norton et al. 1973； Hiên and Navarro-Delmasure 1991; Zhang et al. 2010). Plastome phylogeny of Apocynaceae has been established that based on the largest sampling of molecular characters (Fishbein et al. 2018). However, the data for *C. manghas* is still lacking. Here we provide the chloroplast genome of *C. manghas* for the first time.

We obtained fresh leaves of *C. manghas* from South China Agricultural University (113°20′42.23″E, 23°9′32.66″N) and the specimen (voucher GZ708) was deposited at the South China Agricultural University Herbarium (CANT). We used Plastid Genome Annotator (PGA) (Qu et al. 2019) for chloroplast gene annotation and further correction in the Geneious Prime 2019 (https://www.geneious.com) and submitted them to GenBank (ID:MT527963).

The size of a chloroplast genome of *C. manghas* is 154,428 bp, including a large single copy (LSC, 85,138 bp) region, a small single copy (SSC, 17,390 bp) region and two inverted repeat (IR, 25,950 bp) regions. The circular genome contains 115 genes, including 81 unique protein-coding genes, 4 unique rRNA genes and 30 unique tRNA genes. The overall A-T content of the circular genome is 62.0%, while the corresponding values of the LSC, SSC, and IR regions are 64.0%, 67.7%, and 56.9%, respectively.

18 chloroplast genomes were selected for their phylogenetic establishment using the maximum likelihood (ML) criterion in IQ-TREE (Nguyen et al. 2015). Two species, *Gentiana crassicaulis* (Gentianaceae) and *Coffea arabica* (Rubiaceae), were used as outgroups. The results support *C.manghas* in Plumerieae, and it is close to *Thevetia peruviana* (Figure 1). The chloroplast genome provides a resource for the phylogenetic studies of Apocynaceae.

**Figure 1.**
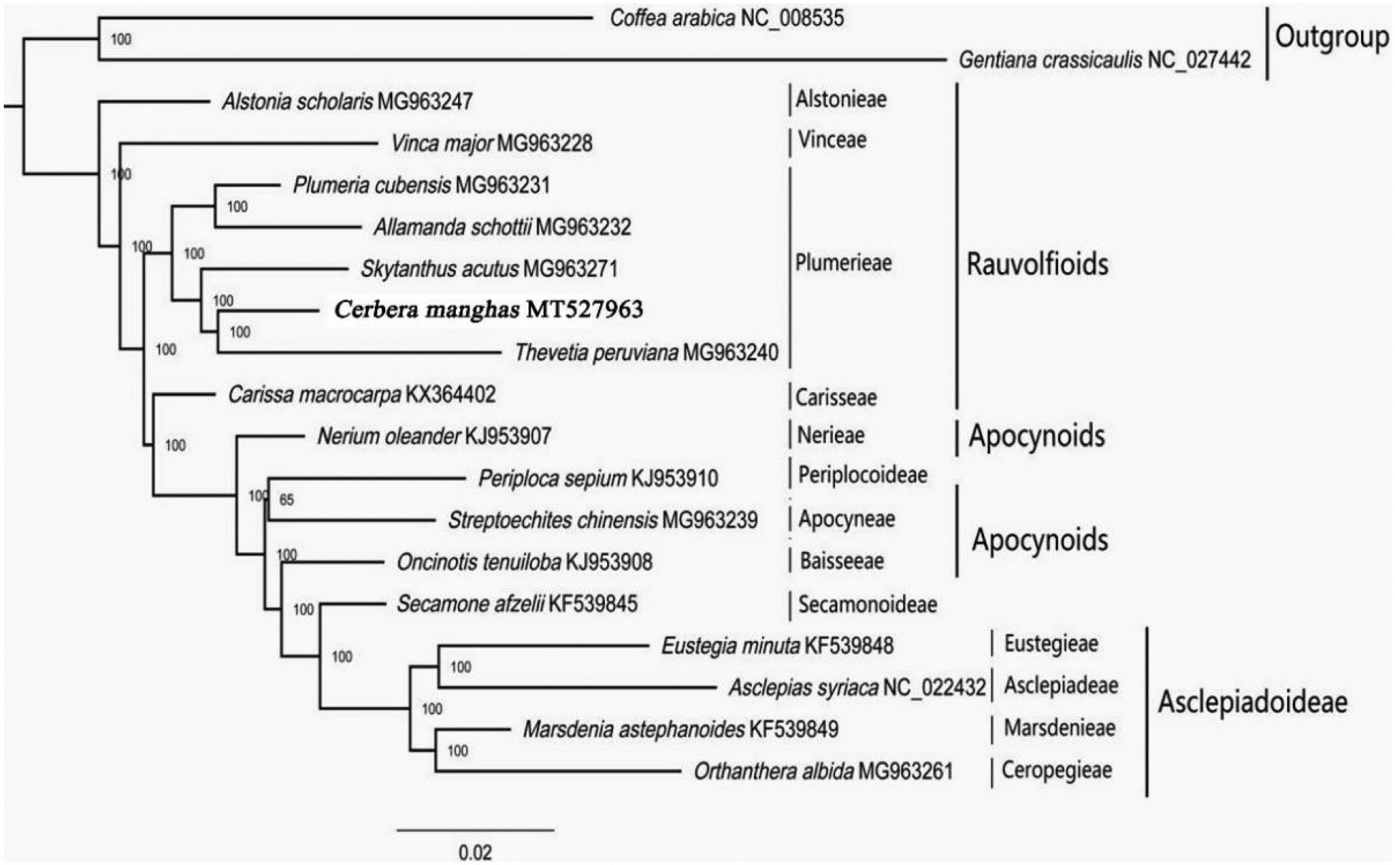
ML phylogenetic tree of *C. manghas* with 18 species was constructed by chloroplast plastome sequences. Numbers on branches are boot strap support values. (The division of tribes and subfamilies refers to the (Endress et al. 2018)).

## Data Availability

The data that support the findings of this study are openly available in NCBI at https://www.ncbi.nlm.nih.gov/, reference number is MT527963.
